# Exploring Skin Wound Healing Models and the Impact of Natural Lipids on the Healing Process

**DOI:** 10.3390/ijms25073790

**Published:** 2024-03-28

**Authors:** Vivek Choudhary, Mrunal Choudhary, Wendy B. Bollag

**Affiliations:** 1 Department of Physiology, Medical College of Georgia, Augusta University, Augusta, GA 30912, USA; vchoudhary@augusta.edu (V.C.);; 2 Charlie Norwood VA Medical Center, Augusta, GA 30904, USA; 3 Department of Dermatology, Medical College of Georgia, Augusta University, Augusta, GA 30912, USA; 4 Department of Medicine, Medical College of Georgia, Augusta University, Augusta, GA 30912, USA

**Keywords:** cutaneous wound healing, lipid, inflammation, keratinocytes, skin, phosphatidylglycerol, in vivo models, epidermal regeneration

## Abstract

Cutaneous wound healing is a complex biological process involving a series of well-coordinated events aimed at restoring skin integrity and function. Various experimental models have been developed to study the mechanisms underlying skin wound repair and to evaluate potential therapeutic interventions. This review explores the diverse array of skin wound healing models utilized in research, ranging from rodent excisional wounds to advanced tissue engineering constructs and microfluidic platforms. More importantly, the influence of lipids on the wound healing process is examined, emphasizing their role in enhancing barrier function restoration, modulating inflammation, promoting cell proliferation, and promoting remodeling. Lipids, such as phospholipids, sphingolipids, and ceramides, play crucial roles in membrane structure, cell signaling, and tissue repair. Understanding the interplay between lipids and the wound microenvironment provides valuable insights into the development of novel therapeutic strategies for promoting efficient wound healing and tissue regeneration. This review highlights the significance of investigating skin wound healing models and elucidating the intricate involvement of lipids in the healing process, offering potential avenues for improving clinical outcomes in wound management.

## 1. Introduction

Skin wound healing is a complex and intricate biological process essential for the restoration of tissue integrity and functionality. The ability of the skin to efficiently heal wounds is critical for maintaining homeostasis and protecting the body from infections and other external threats. Therefore, understanding the mechanisms underlying this process is of paramount importance, allowing for the development of effective therapeutic strategies to enhance wound healing and the improvement of patient outcomes. In this review, we briefly discuss the landscape of various skin wound healing models, focusing on models of full-thickness excisional skin wounds, rather than burn or incisional wounds or other penetrating injuries. Full-thickness excisional wounds offer distinct advantages in terms of reproducibility, ease of creation, and relevance to clinical scenarios. By concentrating on this specific type of wound model, we aim to streamline the discussion and provide a clear framework for researchers to select the most appropriate model for their studies. Each model serves as a unique lens through which we gain insights into the multifaceted mechanisms at play during the wound healing process. We also discuss the advantages and disadvantages of the various options so that researchers can select the best model to address their question of interest. 

We further focus on the influence of natural lipids in orchestrating and modulating cutaneous wound healing. Lipids are essential components of the skin barrier and play crucial roles in maintaining skin integrity and function. Emerging evidence suggests that lipids are not only passive structural elements, but also actively participate in the regulation of various aspects of wound healing, including inflammation, cell migration, proliferation, and tissue remodeling. By elucidating the roles of natural lipids in orchestrating and modulating cutaneous wound healing, we aim to provide insights into the complex interplay between lipid metabolism and tissue repair processes. The impact of lipids on the healing process introduces a dynamic dimension, adding layers of complexity to our comprehension of how skin restores itself after injury. By uncovering the molecular mechanisms underlying the effects of lipids on wound healing, we can identify novel therapeutic targets and develop innovative interventions to promote the more efficient and effective healing of cutaneous wounds. Thus, a better understanding of the mechanisms underlying proper wound healing should allow for the identification of pathways, including lipid pathways, that can be targeted for effective therapies to improve skin wound healing.

In summary, this review article aims to provide readers with a comprehensive overview of skin wound healing models and the impact of natural lipids on the healing process. By synthesizing existing knowledge and highlighting key findings, we hope to contribute to the advancement of research in this important field and pave the way for the development of novel therapeutic strategies to enhance wound healing and improve patient care.

## 2. Skin Structure

In order to delve more deeply into the complicated terrain surrounding cutaneous wound healing, it becomes necessary to understand the fundamental structure and function of the skin. The skin, the largest organ in the human body, is an intricate structure composed of three main layers: the epidermis, dermis, and hypodermis (subcutaneous tissue). Each layer plays a distinct role in maintaining the skin’s integrity, and together, they contribute to the overall function of the skin, including its ability to regenerate after wounding.

### 2.1. Epidermis

**Figure 1:**
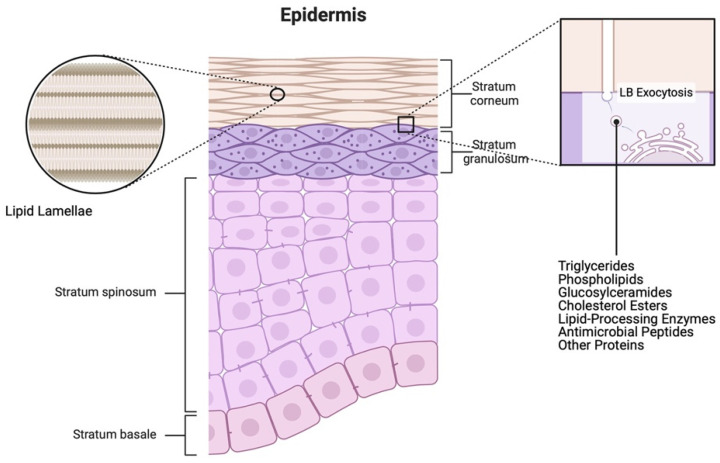
Schematics illustrating the structure of the epidermis and the lamellar body/lipid secretion process. The epidermis primarily consists of keratinocytes arranged in layers. Proliferating keratinocytes reside in the stratum basale, followed by suprabasal keratinocytes in the stratum spinosum, lamellar body (LB)-secreting keratinocytes in the stratum granulosum, and terminally differentiated (dead) keratinocytes (squames or corneocytes) in the stratum corneum. The square inset on the right depicts the composition and exocytosis of LB by differentiating keratinocytes in the stratum granulosum. Lipids secreted via LBs contribute to the formation of lipid lamellae surrounding the corneocytes, establishing the skin’s lipid barrier, as illustrated in the left circular inset. Created with Biorender.com.

The epidermis, the outermost layer of the skin, is a stratified epithelium consisting of melanocytes, Langerhans cells, Merkel cells, and the predominant cell type, keratinocytes [1]. It is a multilayered structure formed by the progressive proliferation and differentiation of keratinocytes, which move from the deep basal layer adjacent to the basement membrane to the upper differentiating spinous, granular, and cornified layers (reviewed in [2]). Each layer exhibits a distinct morphology and specific gene/protein expression pattern contributing to the development of epidermal structure and the permeability barrier. The permeability barrier function of the skin is primarily mediated by the outermost layer, the stratum corneum, composed of corneocytes and inter-corneocyte lipids released from keratinocytes via lamellar bodies (Figure 1).

The ‘brick and mortar model’ proposed by Elias [3] describes the stratum corneum as flat cells (bricks) surrounded by a lipid matrix (mortar). Corneocytes, as terminally differentiated cells, comprise the structure of the stratum corneum and serve as hydrating reservoirs for enzymatic processes. The lipids that the granular layer of keratinocytes synthesize and release as lamellar bodies include phospholipids, triglycerides, glucosylceramides, and cholesterol esters. These lipids serve as precursors for the lipids that eventually form the permeability barrier, fatty acids, ceramides, and cholesterol, which are produced by lipid-metabolizing enzymes that are contained within the secreted lamellar bodies. All of these lipids, in the correct ratio, are key for an efficient epidermal permeability barrier, although ceramides comprise the largest proportion of lamellar lipids. Indeed, there is an incredible array of ceramides, including some specialized ones, such as omega-O-acylceramides, which are unique to the outer layers of the epidermis, and a variety of very-long-chain fatty acids, which can be incorporated into the lamellar ceramides and acylceramides. The backbone of these ceramides can also be one of several different sphingoid bases, including sphingosine, phytosphingosine, and dihydrosphingosine, leading to an incredible variety in the ceramides found in the skin. By synthesizing these lipids, keratinocytes thus play a pivotal role in the skin’s water permeability barrier function, the most important defensive function for terrestrial life, since it impedes the transcutaneous movement of water and other essential electrolytes [4].

Keratinocytes derive their name from the presence of the abundant cytoskeletal protein, keratin. The assembly of keratin monomers generates intermediate filaments, which are involved in several key properties of keratinocytes, including structural integrity, mobility, and metabolic signaling [5]. Keratinocytes also release various effector molecules, such as cytokines, chemokines, anti-microbial peptides, growth factors, and lipid mediators. These secreted factors actively participate in the recruitment of inflammatory cells, highlighting the substantial role played by keratinocytes in maintaining skin immune homeostasis [6], as well as disease [7]. Furthermore, the relationship between keratinocytes, immune cells, and wound healing is crucial, especially during the initial stages. In addition to secreting key cytokines, chemokines, and growth factors, the basal layer of the epidermis contains actively dividing keratinocytes that play a vital role in regenerating the epidermis after injury. Thus, keratinocytes migrate to cover the wound site, forming a protective barrier against pathogens and reducing the risk of infection during the wound healing process [8].

Langerhans cells are also involved in the immune response, acting as antigen-presenting cells. In an interesting recent study, Wasko and coworkers examined single-cell RNA-sequencing data, revealing cellular interactions in skin repair, mapping the early angiogenic niche in skin wounds, and highlighting the pivotal role of Langerhans cells as regulators of angiogenesis during the skin repair process [9]. These authors utilized lineage-driven reporters, three-dimensional (3D) microscopy, and mouse genetics to reveal that Langerhans cells are positioned at the leading edge of endothelial cells in mouse skin wounds, emphasizing their essential role in angiogenesis during the repair process.

Melanocytes are primarily responsible for the synthesis of melanin, the pigment that contributes to skin color and provides protection against UV radiation [10]. Contrary to the traditional view of melanocytes as less significant in the initial stages of wound healing, recent findings from Gupta et al. (2023) highlight their influence. Melanin-rich cells in pigmented guinea pig skin enhance wound healing, appearing during the proliferative phase. Moreover, melanocyte-conditioned media, containing transforming growth factor-beta (TGFβ), actively promotes keratinocyte migration [11]. In a separate study, the intraperitoneal injection of alpha-melanocyte-stimulating hormone before the creation of excisional skin lesions inhibited leukocyte infiltration into the wound, resulting in improved wound healing [12]. Melanocytes can produce various cytokines and growth factors [13,14] that may have an impact on the inflammatory response, influencing the overall wound healing process. Melanocytes may also play a role in scar formation. Thus, melanocytes have been shown to stimulate the growth and proliferation of fibroblasts, to enhance collagen synthesis, and to promote extracellular matrix deposition. Additionally, they can activate the TGFβ signaling pathway, potentially contributing to the development of pathological scarring [15]. It is important to note that the understanding of melanocytes’ role in wound healing is still an evolving area of research, and the precise mechanisms are not fully elucidated. Further studies are needed to comprehensively understand the contributions of melanocytes in different stages of the wound healing process.

Merkel cells function as mechanoreceptors involved in touch sensation [16]. There is speculation about the potential contribution of Merkel cells to wound repair in terms of engaging in sensory aspects of the healing process, such as potentially influencing cell migration and tissue remodeling [17]. However, a comprehensive understanding of the extent of Merkel cells’ involvement in wound healing and the underlying mechanisms requires further research.

### 2.2. Dermis

Beneath the epidermis lies the dermis, a connective tissue layer rich in blood vessels, nerves, and various cell types [16]. Fibroblasts serve as the primary cells in the dermis, with myofibroblasts, mast cells, macrophages, and endothelial cells comprising crucial cellular components within the dermal structure. Fibroblasts are key contributors to wound healing. They produce collagen and other extracellular matrix (ECM) components, providing structural support for tissue repair [18]. Myofibroblasts are specialized fibroblasts that contribute to wound contraction. They play a crucial role in reducing the wound size during the remodeling phase. Mast cells release various mediators, such as histamine and growth factors, which influence inflammation and tissue repair. They play a role in the initial phases of the healing process, and recent studies have emphasized the pivotal role of mast cells in influencing the degree of scar tissue formation in the course of wound healing [19]. Macrophages, as part of the immune system, contribute to the removal of debris, to pathogen defense, and to the promotion of tissue repair [20]. They play a crucial role in the inflammatory and proliferative phases of wound healing. Endothelial cells contribute to angiogenesis, the formation of new blood vessels. This process is thought to be essential for supplying nutrients and oxygen to the healing tissue during the proliferative phase. Although neutrophils are not frequently observed in normal skin, they are recruited in high numbers as early responders to injury and are involved in the initial inflammatory phase [21]. They help control infections and clear debris from the wound site.

### 2.3. Subcutaneous Tissue

The subcutaneous tissue, also known as the hypodermis, is located beneath the dermis and is primarily composed of adipocytes (fat cells) and connective tissue. Adipocytes provide thermal insulation and energy storage, and their presence can influence wound healing indirectly by affecting overall skin health. The subcutaneous tissue also contains nerves and blood vessels that contribute to supplying nutrients and oxygen to the surrounding tissues, indirectly supporting the healing process in the skin [22]. Adipose tissue releases cytokines and growth factors that modulate the immune response and influence the overall healing process.

These intricate skin layers form the foundation for the remarkable regenerative capacities of this organ during the wound healing process. Skin structural elements not only provide protection, but also play a crucial role in managing the complex series of events required for effective repair. Examining the functions of the skin in this context unveils its dynamic nature, showcasing how its innate abilities come to the forefront when faced with the challenge of healing wounds. In the upcoming sections, we will discuss the interplay between the skin’s structure and its functions, unraveling the intricacies that contribute to the remarkable phenomenon of cutaneous wound healing.

## 3. Skin Function

The skin, as the body’s largest organ, performs a myriad of functions crucial for maintaining homeostasis, protecting against external threats, and contributing to overall well-being. Each function is intricately linked to the skin’s structure and cellular components, which play a vital role in processes such as wound healing.

### 3.1. Skin as a Protective Barrier

One of the primary functions of the skin is to serve as a protective barrier against environmental hazards, pathogens, and physical injury. The outermost layer of the skin, the epidermis, with its keratinized cells, acts as a formidable shield preventing microbial invasion [16]. This protective function is compromised after wounding, but is restored upon wound healing as the intact skin acts as a barrier, reducing the risk of infection once the healing process is complete. The skin also serves as a barrier to retain important body components, such as water. Indeed, the epidermal water permeability barrier is essential for terrestrial life, and the mutation or deficiency of gene-encoding enzymes involved in the synthesis of permeability barrier lipids can result in perinatal lethality, at least in part, due to excessive water loss and dehydration (for example, [23]).

### 3.2. Skin as a Thermoregulator

The skin plays a crucial role in thermoregulation, helping the body maintain a stable internal temperature. Sweat glands in the skin release perspiration, which evaporates to cool the body. In addition, blood vessels in the dermis regulate heat exchange by vasodilation and vasoconstriction.

### 3.3. Skin as a Sensory Organ

The skin houses numerous sensory receptors that enable the perception of external stimuli such as touch, pressure, temperature, and pain. Nerve endings in the skin’s dermis contribute to tactile sensations and play a vital role in maintaining spatial awareness [16]. Sensory perception is crucial during wound healing, as pain and touch sensations inform the individual about the condition of the wound, prompting protective behaviors and facilitating appropriate care.

### 3.4. Skin as the Frontline of Defense

The skin serves as an integral component of the body’s immune defense system. Langerhans cells in the epidermis act as antigen-presenting cells, initiating immune responses against pathogens [24]. In wound healing, the immune response is vital during the inflammatory phase. Immune cells, including macrophages, migrate to the wound site, clearing debris and initiating the healing process.

### 3.5. Skin as a Mediator of the Synthesis of Vitamin D

Exposure to sunlight allows the skin to produce vitamin D, a crucial factor in calcium absorption and bone health. UV radiation triggers the conversion of 7-dehydrocholesterol to vitamin D in the skin [25,26]. This process is important both for systemic effects of vitamin D, for example, on bone, but also paracrine effects within the skin. Indeed, vitamin D’s pivotal role in wound healing and skin health is underscored by various research findings from multiple groups. Our earlier work shed light on vitamin D’s impact on keratinocyte proliferation, revealing a biphasic effect of active vitamin D, that is, 1,25-dihydroxyvitamin D_3_ [1,25(OH)_2_D_3_] on primary mouse epidermal keratinocytes [27]. We observed that physiological concentrations of 1,25(OH)_2_D_3_ stimulate keratinocyte proliferation, while higher doses inhibit growth. Recently, the Zhang laboratory conducted a clinical trial demonstrating the efficacy of topical vitamin D_3_ in treating chloasma, a condition of hypermelanosis occurring during pregnancy, as well as in enhancing wound healing outcomes [28]. In another study, the Watsky laboratory found that both topical 1,25(OH)_2_D_3_ and 24,25(OH)_2_D_3_ accelerate corneal wound healing in mice [29]. Additionally, in a recent review, Bikle emphasized the importance of vitamin D and calcium signaling in epidermal wound healing, highlighting their role in stem cell activation and re-epithelialization [30]. Collectively, these investigations underscore the critical role of vitamin D in promoting wound healing and improving skin barrier function.

In summary, the functions of the skin are diverse and interconnected, contributing significantly to the body’s overall health and functionality. Understanding these functions provides insights into the intricate role of the skin in processes such as wound healing, emphasizing the holistic nature of the body’s responses to external and internal challenges.

## 4. The Process of Cutaneous Wound Healing

Understanding the nuances of the complex wound healing process not only provides insights into fundamental biology, but also holds promise for developing innovative therapeutic strategies to optimize wound healing outcomes in various clinical scenarios. Cutaneous wound healing is a highly organized and dynamic process involving a series of interconnected and overlapping phases [31,32]. Each phase is characterized by specific cellular and molecular events that collectively contribute to the restoration of tissue integrity.

### 4.1. Hemostasis

The initial phase of cutaneous wound healing is hemostasis, where the goal is to stop bleeding and establish a provisional matrix. Platelets adhere to the exposed collagen at the wound site, initiating the formation of a blood clot [33,34]. Aggregated platelets undergo degranulation, releasing growth factors and chemotactic factors like transforming growth factors (TGFs), platelet-derived growth factor (PDGF), and platelet factor 4, providing a foundation for subsequent events in the wound healing process [35].

### 4.2. Inflammation

Following hemostasis and with the release of chemotactic factors, the inflammatory phase begins, characterized by the infiltration of immune cells and the removal of debris [36]. Neutrophils are the first responders, phagocytosing bacteria and damaged tissue, and later, macrophages play a crucial role in clearing cellular debris and modulating the immune response [37]. Inflammatory cytokines and growth factors coordinate cell migration, angiogenesis, and the initiation of tissue repair. The quality and duration of the inflammatory response significantly influence the wound healing progress. Prolonged inflammation can impede healing and lead to the development of chronic ulcers [38].

### 4.3. Proliferation

The proliferation phase allows the formation of granulation tissue as well as tissue regeneration. Fibroblasts migrate to the wound site, producing collagen, the main component of the ECM, which forms the structural framework of the healing tissue [18,39]. Endothelial cells promote angiogenesis, ensuring the establishment of a robust vascular network to support the growing tissue. Keratinocytes at the wound edges proliferate and migrate, closing the wound through re-epithelialization [40]. Growth factors, including TGFβ and PDGF, orchestrate these cellular activities, stimulating the migration, proliferation, and synthesis of ECM components [41].

### 4.4. Remodeling

The final phase of cutaneous wound healing is remodeling, where the provisional matrix is replaced by a more organized and functional scar tissue. Collagen fibers undergo realignment and maturation, and excess cells undergo apoptosis [39]. The tissue’s tensile strength increases as collagen cross-linking occurs, contributing to the scar’s stability. While the remodeling phase can last for an extended period, maintaining a crucial balance between the actions of matrix metalloproteinases (MMPs) and tissue inhibitors of metalloproteinases (TIMPs) is essential for effective wound repair and remodeling [42,43].

**Figure 2:**
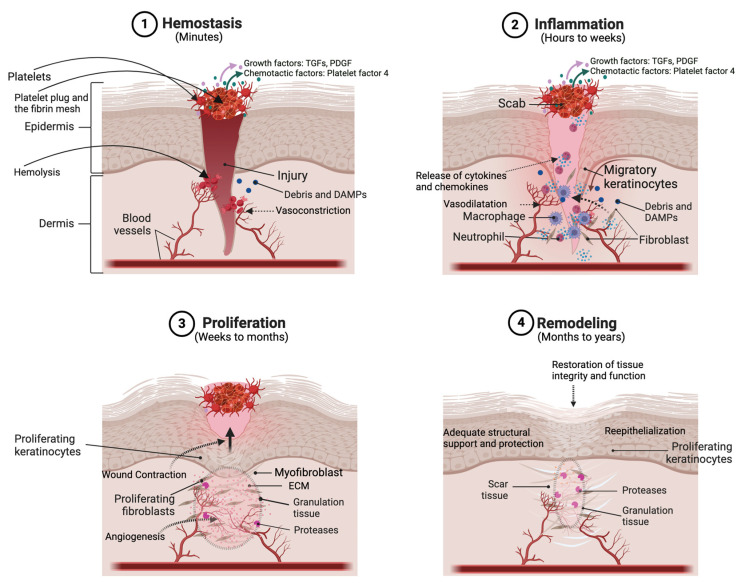
Schematic illustrating the various phases of the wound healing process. A full-thickness injury to the skin wounds the epithelium and dermis, leading to bleeding from damaged dermal blood vessels. (1) In the first phase of wound healing, which occurs within minutes, bleeding is controlled and stopped (i.e., hemostasis is induced) via the formation of a scab composed of adhering platelets and a fibrin mesh. The injury can also result in the release of various growth factors [like transforming growth factors (TGFs) and platelet-derived growth factor (PDGF)] and chemotactic factors, as well as damage-associated molecular patterns (DAMPs). (2) In the inflammation phase, keratinocytes begin to migrate from the wound edge to re-epithelialize the wound. Also, innate immune cells (such as macrophages and neutrophils) are activated, often by microbial components entering the skin through the compromised barrier or DAMPs produced as a result of the injury, to secrete cytokines and chemokines. In addition, fibroblasts are recruited to begin synthesizing extracellular matrix (ECM) proteins. (3) In the proliferation phase, keratinocytes divide to restore the epidermal thickness and dermal fibroblasts/myofibroblasts proliferate and deposit extracellular matrix (ECM) proteins, forming granulation tissue, to regenerate the dermis. Endothelial cells promote new vessel formation (angiogenesis) to ensure adequate blood flow and nutrient supply. (4) During remodeling, collagen fibers mature and become more organized and cross-linked. Excess cells undergo apoptosis and ECM proteins are deposited and degraded in a balanced manner to restore proper tissue architecture and function. Created with Biorender.com.

In conclusion, the wound healing process is a precisely harmonized symphony of cellular and molecular events, involving multiple phases, cellular players, signaling molecules, and dynamic ECM remodeling (see Figure 2). From the rapid and inflammatory response to the proliferative phase and the intricacies of ECM remodeling, each facet of wound healing contributes to the restoration of tissue integrity. The scientific understanding of these phases has been shaped by extensive research, and the intricacies of cutaneous wound healing continue to be explored.

## 5. In Vitro Models of Skin Wound Healing

In vitro models play a pivotal role in unraveling the intricacies of skin wound healing, offering a controlled environment for studying cellular behaviors and molecular processes. These models, ranging from single-cell-type two-dimensional (2D) cultures to advanced three-dimensional (3D) systems (Table 1), provide valuable insights into various aspects of wound repair, enabling researchers to address specific mechanistic questions related to tissue regeneration.

### 5.1. Single-Cell Models

Single-cell-type 2D models have been fundamental in investigating basic cell signaling responses to injury and stress. Typically, relevant cells, such as dermal fibroblasts or keratinocytes, are grown in vitro, and a simulated “wound” is created by scraping the confluent cell layer (“scratch wounding”). This process induces cellular trauma, initiating a cascade of events, including proliferation, protein production, and changes in viability, migration, gene expression, and differentiation, and opens an area that must be covered and closed. Single-cell-type models are frequently employed to test agents that may enhance skin cell migration and re-epithelialization [44,45].

### 5.2. Co-Culture System

Co-culture systems, such as indirect co-cultures using Transwell systems, have facilitated the study of interactions between keratinocytes and fibroblasts. The Transwell migration assay, based on two chambers separated by a porous membrane, allows the analysis of paracrine factors and chemotactic responses for various cell types. These methods contribute to understanding keratinocyte–fibroblast interactions and chemotaxis [41,46].

### 5.3. Three-Dimensional In Vitro Models

In acknowledging the limitations inherent in single-cell-type or co-culture 2D models, researchers are increasingly turning to 3D in vitro models as a more comprehensive approach to replicate the physiological complexity of skin tissue. Utilizing techniques such as incorporating fibroblasts into a type-I collagen matrix provides a means to assess cell contraction and matrix compaction. Another approach involves embedding fibroblasts within a 3D collagen construct, facilitating the observation of cell migration from a denser collagen matrix into the surrounding matrix. Despite advancements in achieving greater physiological relevance with these models, there are ongoing challenges in accurately representing the diverse array of ECM proteins and multiple cell types found in native skin [43].

### 5.4. Three-Dimensional Skin Equivalents

Advancing beyond traditional monolayer cultures, 3D skin equivalents, also known as reconstructed human epidermis, represent a significant leap in mimicking the complex architecture of native skin. These models incorporate multiple cell types and layers, including keratinocytes, fibroblasts, and sometimes immune cells, closely recapitulating the microenvironment of a healing wound. The inclusion of ECM components in these models enhances their physiological relevance, making them valuable tools for studying aspects such as tissue regeneration and re-epithelialization [47,48].

### 5.5. Three-Dimensional Bioprinting

In addressing challenges related to skin structure restoration, 3D bioprinting emerges as a promising solution for constructing patient-specific skin grafts with biomimetic structures, showcasing potential improvements in regeneration as well as clinical outcomes in wound healing. For a concise overview of recent advances in skin tissue engineering through the application of 3D bioprinting, please consult reviews by Antezana et al. and Zhang et al. [49,50].

### 5.6. Microfluidic Platforms

Over the past two decades, microfluidic wound healing assays have evolved, employing various microchannel designs for creating cell-free areas through cell exclusion or depletion. Cell depletion methods, utilizing thermal, electric, enzymatic, or mechanical principles, remove cell subpopulations to generate cell-free wound areas. On the other hand, cell exclusion assays initially prevent cell adhesion on specific substrate portions, creating wound defects upon the removal of the cell-blocking structure. These microfluidic platforms provide a unique capability to analyze molecular processes in wound healing, studying cell migration, cell–cell interactions, and even skin inflammation models-on-a-chip. Overall, microfluidic systems offer a versatile and sophisticated approach to advancing our understanding of wound healing processes [51,52].

### 5.7. Advantages and Limitations of In Vitro Models

In vitro models present numerous advantages in the exploration of skin wound healing. They offer a controlled environment conducive to the dissection of specific cellular responses and interactions, enabling a meticulous examination of the molecular pathways involved in various phases of wound repair. Moreover, these models stand as a cost-effective and ethical alternative to in vivo studies, mitigating the need for extensive animal experimentation. However, it is imperative to recognize the inherent limitations of in vitro models. The simplifications inherent in these systems may not fully capture the dynamic and multifaceted nature of in vivo wound healing. Notably, challenges arise from the absence of the complete spectrum of immune responses and the inability to replicate the intricate complexity of the tissue microenvironment. Acknowledging these limitations is crucial for a comprehensive understanding of research findings and their translational relevance.

## 6. Ex Vivo Models or Skin Explant Culture Models

Ex vivo models of skin wound healing involve the use of living tissue samples taken from an organism and maintained in culture to study the mechanisms and dynamics of wound repair. These models provide a bridge between in vitro cell cultures and in vivo studies, allowing researchers to explore tissue responses to injuries in a more complex and physiologically relevant context. In the “donut-shaped” wound healing model, human skin is excised and excess subcutaneous adipose tissue is removed from the sections. A circular excisional wound is then created within the explant using a circular punch biopsy to produce a sample resembling a doughnut. These skin sections with a circular wound in the middle are cultured to monitor wound healing. These ex vivo models maintain the multicellular and ECM components of intact skin, providing valuable insights into cell interactions, migration, and tissue regeneration. Researchers have utilized skin explant models to investigate diverse aspects of wound healing, such as inflammation, fibroblast activation, and epidermal repair [53,54]. However, they may fail to adequately mimic the gas, nutrient, and hormonal delivery of an in vivo system.

## 7. In Vivo Models of Skin Wound Healing

Wound healing is a complex biological process involving a series of precisely synchronized events aimed at restoring tissue integrity and function. To gain insights into the intricate mechanisms underlying this phenomenon, researchers often turn to animal models.

### 7.1. Rodent Models

#### 7.1.1. Full-Thickness Wound Model

**Figure 3:**
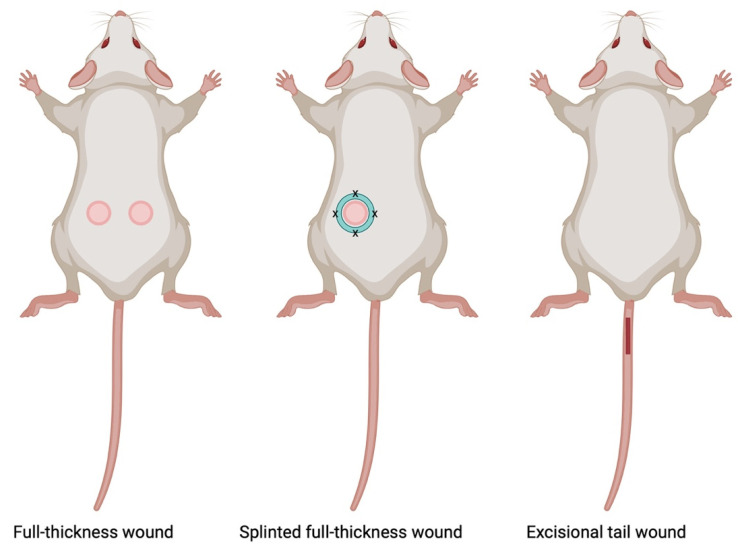
Schematic representation of rodent wound healing models. This illustration depicts common rodent wound healing models involving full-thickness wounds and splinted full-thickness wounds on the dorsal skin of mice, along with tail excisional wounds. Created with Biorender.com.

Rodent models have emerged as valuable tools, offering a balance between physiological relevance and practicality in experimental settings (Figure 3). Full-thickness rodent models involve the creation of wounds that penetrate through the entire thickness of the skin, encompassing both the epidermis and dermis [55,56]. These models replicate key aspects of human wound healing and provide a platform for investigating various facets of tissue repair, including cellular responses, molecular signaling, and the dynamics of extracellular matrix remodeling.

There are several advantages to this model including: (1) physiological relevance in that the full-thickness nature of these models closely mimics human wounds, allowing for a more accurate representation of the healing process. The inclusion of both epidermal and dermal layers facilitates the study of the cell types and interactions critical to wound closure; (2) clinical translatability, as findings from full-thickness rodent models often exhibit translational relevance to human wound healing, enhancing the potential for clinical applicability; and (3) versatility, since researchers have the ability to manipulate various parameters, such as wound size and location, and to conduct studies in transgenic mouse models in order to address specific research questions or simulate different clinical scenarios.

On the other hand, there are certain considerations and limitations of the rodent models, including species-specific variations, such that rodent models exhibit differences in wound healing compared to humans. For example, rodents heal mainly by wound contraction, whereas for humans, re-epithelialization predominates. In addition, rodents generally display more rapid wound healing than humans. Therefore, despite their advantages, the differences in rodent wound healing compared to humans emphasizes the importance of a cautious interpretation and careful consideration when extrapolating the results.

#### 7.1.2. Splinted Full-Thickness Wound Model

The splinted wound model involves the creation of a standardized full-thickness wound, often on the dorsum of rodents, with the application of a splint, generally made of silicone, to minimize wound contraction. The splint serves to mechanically hold the wound open, preventing the natural contraction of the wound edges and forcing healing by re-epithelialization. This model allows researchers to focus on specific aspects of healing without the confounding effects of wound closure by contraction [57,58]. Thus, one of the key advantages of this model is the limit on contraction, enabling the study of granulation tissue formation, re-epithelialization, and ECM deposition, thereby creating a more translational model. Other benefits of the model include the ability to obtain precise measurements and minimize variability—splinting allows researchers to precisely measure wound dimensions and closure rates, facilitating the accurate quantification of healing parameters over time while decreasing the variability associated with natural wound contraction. Both of these advantages lead to more consistent and reproducible experimental outcomes. On the other hand, although the splinted wound rodent model results in healing in a more similar way to human skin, the skin of mice and rats is still quite different from that of humans. Therefore, the results from rodent models may not perfectly mirror human wound healing, necessitating careful interpretation. In addition, the use of the splint, which must be sutured and/or glued in place, introduces an artificial constraint to wound closure, and researchers should be cautious in extrapolating findings to natural healing scenarios.

#### 7.1.3. Tail Excisional Wound Model

The tail excisional model involves the complete removal of skin tissue from the designated area on the dorsal surface of the mouse or rat tail, typically performed as a full-thickness excision [59]. Tail wounds, in contrast to dorsal wounds, typically take up to 21 days for complete resurfacing, making them a suitable model for investigating mechanisms of delayed wound healing [60]. This model is particularly useful for studying wound closure dynamics and re-epithelialization processes in a relatively small and accessible area. The rat tail model shows minimal wound shrinkage and biological traits akin to normotrophic and hypertrophic scars in humans, whether produced with or without stretching [61]. This model holds promise for examining cutaneous wound healing and scarring concurrently. In addition, the tail excisional wound model offers accessibility, reproducibility, and controlled wound parameters for studying wound healing processes in small animal models. However, its limitations include relatively small wound sizes, differences in wound healing dynamics compared to other anatomical sites, and restricted wound complexity. Despite these drawbacks, the model remains valuable for investigating fundamental aspects of tissue repair and regeneration.

#### 7.1.4. Chronic Wound Models

Chronic wounds, characterized by impaired or delayed healing, pose a significant clinical challenge. Chronic or non-healing wounds affect approximately 6.5 million patients in the United States and cost a reported USD 25 billion or more annually in treatment expenses [62]. Animal models of chronic wounds, such as diabetic or aged mice, are employed to mimic the complexities of chronic wound healing, since both diabetes and aging are associated with impaired cutaneous wound healing [63,64,65]. These models allow researchers to investigate the factors contributing to delayed healing, including impaired angiogenesis and persistent inflammation. Understanding these mechanisms is crucial for developing targeted interventions for chronic wounds [66].

A diabetic model of chronic wounds was recently developed using approximately 6-month-old db/db mice which developed diabetes and obesity due to an inactivating mutation in the gene encoding the leptin receptor [67]. For this model, an excisional full-thickness skin wound is made on the shaved, depilated dorsum of obese db/db mice under non-sterile conditions. Indeed, bacterial exposure appears to be important to generate chronicity in the wound of these mice, which should also be housed in a conventional vivarium using non-sterile food and bedding (i.e., not autoclaved) [67]. Upon the generation of the wounds, the mice are treated with oxidative-stress-promoting drugs: they are injected (intraperitoneally) with 3-amino-1,2,4-triazole to inhibit catalase and topical mercaptosuccinic acid to inhibit glutathione peroxidase. The oxidative stress resulting from the use of these drugs induces necrosis of the wound margin [67], whereas the bacterial exposure allows the formation of a biofilm. Biofilms can be observed by 3–10 days after wounding, and the wound is considered chronic if it remains unhealed for more than 20 days after its generation [67]. The model can be examined at different times after wounding to determine the factors involved at various points in the pathway leading to chronicity, and/or it can be used to test therapies for their efficacy in promoting the healing of chronic wounds.

**Figure 4:**
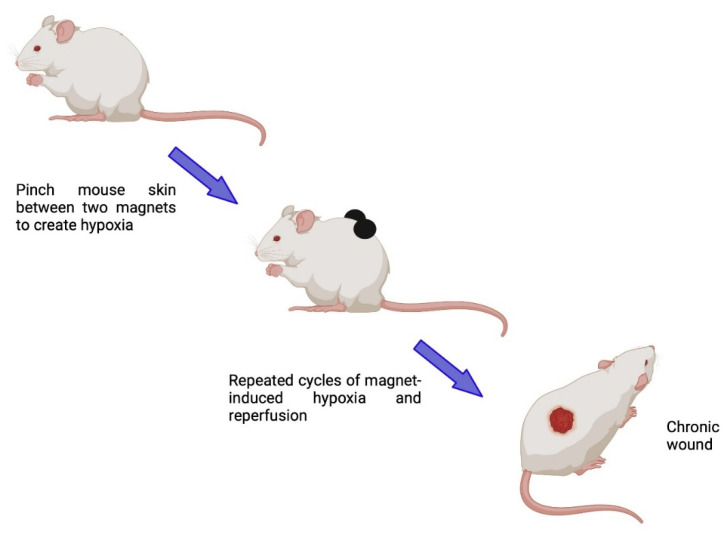
Schematic illustrating the progressive modeling of chronic skin wounds resembling pressure ulcers with repeated cycles of ischemia–reperfusion using magnetic plates to compress the skin. Created with Biorender.com.

In addition to these diabetic and aging models of delayed cutaneous wound healing, one specific model for investigating chronic wounds has been described by Stadler and coworkers, who used magnetic plates to create wounds mimicking pressure ulcers [68] (Figure 4). In this model, folded mouse back skin is placed between two magnetic plates to compress the skin and then the pressure is increased, thereby inhibiting skin perfusion. The magnetic plates are placed on the skin and removed a number of times (every 16 h or so) to produce repeated ischemia–reperfusion cycles that result in the formation of pressure ulcers. The lesions are allowed to reach their maximum (at approximately 10 days post-injury) and their healing can then be tracked over time.

#### 7.1.5. Special Considerations for Use in Conjunction with Different Wound Models

##### Genetically Modified (Transgenic) Models

Advancements in genetic engineering have led to the development of transgenic and knockout animal (particularly mouse) models to study the specific roles of genes and signaling pathways in wound healing. These models allow for the manipulation of genes associated with inflammation, angiogenesis, and extracellular matrix remodeling [69] and are used in conjunction with one of the wounding models described above to determine the possible involvement of the particular manipulated gene in healing.

##### Immunocompromised Model

The immune response plays a critical role in wound healing, and immunocompromised animal models, such as severe combined immunodeficiency (SCID) mice, have been instrumental in studying the impact of the immune system on wound repair. These models allow for the investigation of the roles of immune cells, including macrophages and T cells, in different phases of wound healing [70]. As with genetically specialized mice, the immunodeficient animals are wounded using one of the other wounding models discussed above.

### 7.2. Rabbit Ear Model

**Figure 5:**
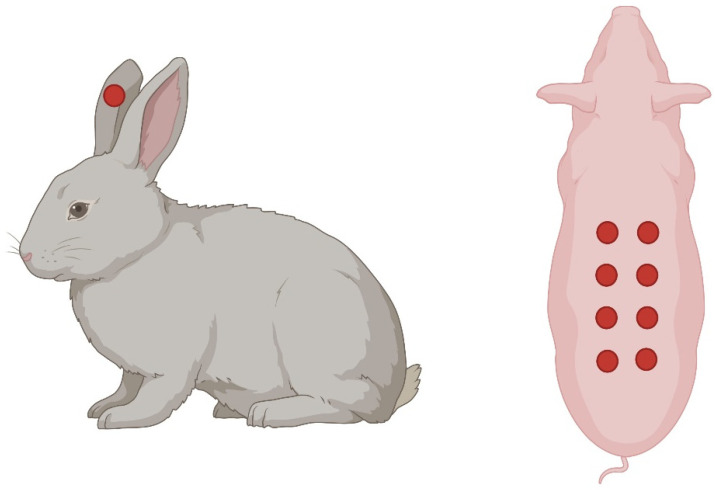
Schematic depicting full-thickness excisional wounds created on the rabbit ear (left) and on the dorsal skin of the pig (right). Each of the red circles represents a full-thickness wound. Created with Biorender.com.

This method involves creating full-thickness hypertrophic scar wounds on the rabbit ear for evaluating the effects of different treatments [71] (Figure 5). While the rabbit ear wound healing model offers several advantages, such as easy access, standardized wound creation, and rapid healing, it also has some limitations. One major drawback is the anatomical and physiological differences between rabbit and human skin. Rabbits have thicker skin with a different composition of collagen fibers and a distinct hair follicle pattern compared to humans. These differences may affect wound healing processes and limit the translatability of findings to human clinical scenarios.

### 7.3. Pig Skin Model

While rodent and rabbit models provide valuable insights into skin wound healing, the translational relevance to humans often requires the use of larger animal models. Porcine models, in particular, share similarities with human skin anatomy and physiology. Full-thickness excisional wounds in pigs allow for a closer representation of the wound healing processes observed in humans (Figure 5). These models enable researchers to explore interventions, such as novel dressings or therapeutics, in a setting more akin to clinical scenarios [72].

### 7.4. Superficial or Tape Stripping Model to Investigate Permeability Repair

**Figure 6:**
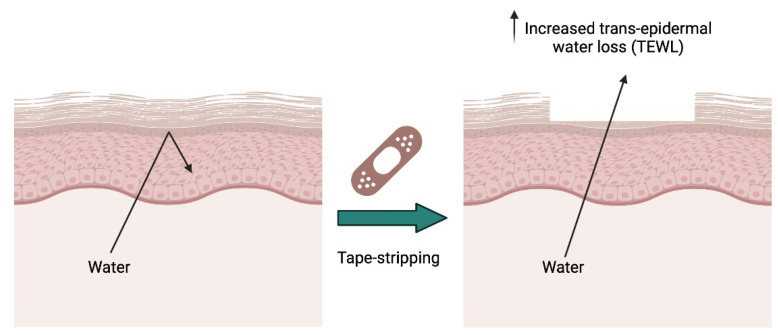
Schematic illustrating the tape stripping model and its ability to disrupt the epidermal water permeability barrier and enhance trans-epidermal water loss (TEWL). Created with Biorender.com.

Although perhaps not a true wound healing model, the superficial or tape stripping model represents a valuable and non-invasive approach to study the “healing” of the permeability barrier of the skin, offering researchers insights into this process (Figure 6).

In this model, a defined area of the skin is subjected to the gentle application and removal of adhesive strips or tape. This process selectively removes the stratum corneum without causing significant damage to the underlying layers, creating a controlled and reproducible superficial wound, i.e., disruption of the epidermal barrier [73]. By mimicking surface injuries, researchers can investigate the mechanisms underlying the dynamics of skin barrier restoration and the accompanying inflammatory responses. The advantages of this model include its non-invasive nature, allowing for repeated sampling and observation without extensive trauma to the skin, its consistency and reproducibility, which enhances the reliability of experimental outcomes, and its focus on the epidermal permeability barrier in that by targeting the stratum corneum, researchers can specifically study skin barrier restoration. The limitations include that damage occurs only to the upper epidermal layers, providing insights into barrier repair, but not deeper tissue wound healing processes, and possible species-specific differences that should be considered since they can affect the ability to extrapolate the results obtained from rodent models to human skin. Alongside the discussion in the text, a comprehensive comparison of in vivo models for cutaneous wound healing studies is presented in Table 2. This table provides a succinct overview of various experimental models used in wound healing research, highlighting their respective advantages and limitations.

In conclusion, the choice of animal model depends on the specific research objectives and the aspects of wound healing being studied. For studies focusing on fundamental molecular mechanisms, genetically modified rodent models may be preferred due to the ability to target specific pathways. When investigating wound closure dynamics and early-stage healing processes, rodent models, especially the full-thickness splinted wound model, offer valuable insights. For research aiming to closely mimic human wound healing, pig skin models may provide the most relevant anatomical and physiological characteristics. However, researchers must also consider factors such as cost, availability, ethical issues, and the translational potential of findings when selecting the most appropriate model for their studies.

## 8. Lipid Signals in Wound Healing

Lipids play many roles in the skin. In the epidermis, in addition to the lipids that comprise the lamellae of the permeability barrier, there are the lipids that form the lipid bilayer of the cell and those, such as fatty acids, that are used as fuel. In addition, the sebaceous glands produce sebum, which plays important roles in the waterproofing of the skin and hair, thermoregulation, and photoprotection [74]. Sebum is composed of a variety of lipids, including the major ones, triglycerides, and fatty acids; however, there are also a few unusual lipids, for example, the wax esters, which are particularly good for waterproofing, and squalene, which is thought to act as an antioxidant in the skin. In the dermis, there are the membrane and signaling lipids, as well as fuel lipids stored in depots in the dermal adipocytes and in the subcutaneous fat of the hypodermis. In addition, lipids also serve as signaling molecules to regulate skin homeostasis, including wound healing.

Wound healing is a complex process that involves many molecular signals. Once viewed primarily as structural components of cell membranes, lipid molecules have emerged as key signaling entities, and in the subsequent sections, we focus on lipid signals. Lipids play a crucial role in the wound healing process by modulating inflammation, angiogenesis, proliferation, and tissue repair. This review explores the diverse roles of lipid signals in different phases of wound healing and their potential therapeutic implications.

### 8.1. Lipids in Barrier Restoration and Skin Hydration

Lipids are essential for the formation and maintenance of the epidermal barrier as well as for hydration by preventing transepidermal water loss; thus, lipids play a vital role in the protection of the skin against external insults and retaining vital components within the body [75,76,77,78]. Lipid species on the body’s surface exhibit antimicrobial activity and play a direct role in shaping the commensal microbiota [78]. The stratum corneum’s intercellular spaces require a distinctive blend of lipids, with ceramides, a type of sphingolipid, constituting approximately half of these intercellular lipids [79]. Free fatty acids maintain low pH levels, which hinder the growth of pathogenic microorganisms and boost skin immunity by stimulating the expression of human β-defensin 2, a key antimicrobial peptide in human skin [80].

### 8.2. Lipids as Mediators of Inflammation

In the initial stages of wound healing, inflammation serves as a protective response to clear debris, prevent infection, and initiate the repair process [37,81]. Several lipids are known to be pro-inflammatory. For example, prostaglandins, a subset of eicosanoids including the prostaglandin derivative thromboxane A2 (TXA2), play a role in vasodilation, fever, and pain during inflammation. TXA2 is mainly produced by activated platelets in healing wounds, helping to amplify platelet activation and irreversibly aggregate platelets for hemostasis [82]. TXA2 receptor deficiency leads to prolonged bleeding in mice [83], and a TXA2 antagonist increases the bleeding time in humans [84]. In a murine model of cutaneous inflammation, TXA2 generated by activated platelets was observed to stimulate the production of the pro-inflammatory cytokine interleukin (IL)-6 and prostaglandin E_2_ (PGE_2_), while concurrently inhibiting the expression of the anti-inflammatory macrophage marker CD206 in the macrophages. These effects were mediated by the activation of the thromboxane-prostanoid receptor [85]. Pro-inflammatory prostaglandins, such as PGE_2_, are produced in macrophages by the action of cyclooxygenase enzymes [86] and contribute to vasodilation and increased vascular permeability, facilitating immune cell recruitment to the wound site [87] and activating phagocytosis [88].

Leukotrienes (LTs) are lipid molecules produced by various cells, including leukocytes, mast cells, and macrophages, in response to inflammation and immune system activation. LTs such as LTB_4_, LTC_4_, LTD_4_, and LTE_4_ are synthesized from LTA_4_, which is derived from arachidonic acid metabolism via the action of 5-lipoxygenase. LTs play a vital role as potent chemoattractants and help the body fight infections and heal wounds by bringing in white blood cells to the affected area. They are also involved in skin disorders and can affect how wounds heal. The excessive production of LTB_4_ can lead to uncontrolled neutrophil chemotaxis and impaired wound healing in diabetic mice [89]. Researchers found that inhibiting or ablating the gene encoding 5-lipoxygenase to reduce LT production can enhance wound healing [90,91,92], suggesting that these lipid signaling molecules are detrimental to optimal healing.

Cholesterol, another crucial lipid, influences wound healing by modulating inflammation and tissue repair [77]. It participates in the synthesis of steroid hormones, like glucocorticoids, which act as anti-inflammatory agents and suppress the immune response [93]. Moreover, cholesterol is involved in the organization of lipid rafts, which play a role in cell signaling during wound closure [94]. Sphingolipids also play multifaceted roles in the wound healing process. Sphingosine-1-phosphate (S1P) is a bioactive lipid molecule that promotes inflammation by regulating immune cell trafficking, vascular permeability, and cytokine production [95]. Another sphingolipid, ceramide-1-phosphate (C1P), is a bioactive lipid molecule with diverse roles in cellular signaling and inflammation. C1P mediates inflammation by stimulating cytosolic phospholipase A_2_, leading to the release of arachidonic acid and subsequent prostaglandin formation, contributing to the inflammatory response and tissue repair processes [96].

Omega-3 fatty acids, including eicosapentaenoic acid (EPA) and docosahexaenoic acid (DHA), have a complex role in modulating the inflammatory response, with mixed reports suggesting both pro-inflammatory and anti-inflammatory effects (see below in Section 8.3). McDaniel and coworkers examined the effects of marine-derived omega-3 fatty acids on pro-inflammatory cytokine production and wound healing in healthy human skin [97]. Their study compared plasma fatty acid levels between two groups of individuals: one receiving omega-3 supplements and the other a placebo. The results showed elevated levels of proinflammatory cytokine IL-1β in the omega-3 group, suggesting a potential pro-inflammatory effect. Additionally, the wound closure time was somewhat longer in the omega-3 group. In another study, rats were fed diets with varying fat compositions before and after wounding [98]: it was found that healed wounds from rats fed omega-3-rich diets were weaker compared to those from rats fed standard diets. This weakness was attributed to alterations in the fibroblastic or maturational phases of wound healing.

### 8.3. Lipid Mediators in the Resolution of the Immune Response (Inflammation)

The resolution of the immune response, a critical aspect of inflammation, requires the dynamic coordination of lipid mediators that actively contribute to restoring tissue homeostasis. Key specialized pro-resolving lipid mediators (SPMs), such as resolvins, protectins, and maresins, are derived from EPA and DHA and actively dampen inflammation to promote the wound resolution phase [87]. These lipid mediators exert their effects by modulating various cellular processes, including inhibiting neutrophil infiltration, enhancing the macrophage phagocytosis of apoptotic cells, and reducing the production of pro-inflammatory cytokines [99]. By engaging specific receptors and signaling pathways, SPMs contribute to the active resolution of immune responses, ensuring a controlled resolution of inflammation and preventing chronic inflammatory conditions. Sphingolipids, including S1P, have also been implicated in immune cell trafficking, as well as the regulation of vascular integrity (see below), influencing the resolution of inflammation in the context of tissue repair [100].

Contrary to the pro-inflammatory effects described above in Section 8.2, there is evidence suggesting that omega-3 fatty acids can also exhibit anti-inflammatory properties in wound healing. For example, male Wistar rats with wounds on their backs were treated with a topical solution of DHA [101]. This treatment accelerated rat skin wound healing by activating G-protein-coupled receptor 120 (GPR120), which reduced IL-1β expression and increased IL-6 levels, indicating anti-inflammatory effects. Additionally, DHA enhanced TGFβ expression and the keratinocyte differentiation marker, involucrin, promoting tissue repair. In a separate investigation, diabetic male Wistar rats induced by streptozotocin underwent excisional wounds on their dorsal skin and received an intraperitoneal administration of Omegaven, a fish-oil emulsion containing EPA and DHA [102]. This intervention resulted in faster wound healing and improved skin morphometric indices. The conflicting reports on the role of omega-3 fatty acids in wound healing highlight the importance of considering various factors such as the experimental model, wound type, and ratio of fatty acids used in supplementation. These variables may contribute to the observed differences in the outcomes, with some studies showing pro-inflammatory effects while others demonstrate anti-inflammatory properties. Further investigation into the specific mechanisms involved and the optimal conditions for omega-3 fatty acid supplementation is necessary to reconcile these discrepancies and maximize their potential therapeutic benefits in wound healing.

Our findings with the lipid signal, phosphatidylglycerol (PG), indicate that PG derived from egg expedites the process of skin wound healing [103]. Moreover, a specific type of PG, dioleoylphosphatidylglycerol (DOPG), has demonstrated potential in treating skin inflammation in mouse models [104]. Building on these discoveries, our studies highlight that DOPG also plays a role in promoting corneal wound healing [105]. These observations suggest the overarching ability of PG to facilitate epithelial wound healing.

Another phospholipid, platelet-activating factor (PAF), serves a dual role in inflammation [106]. Initially, it triggers inflammation by promoting immune cell recruitment, enhancing leukocyte adhesion and extravasation, stimulating the release of pro-inflammatory mediators, and modulating vascular permeability. However, during the resolution phase, the PAF changes towards resolving inflammation by promoting the apoptosis of neutrophils and the clearance of inflammatory cells and debris from the site of injury, and by regulating pro-inflammatory mediators [106].

Thus, the coordinated resolution of inflammation, facilitated in part by lipid mediators, is intricately linked to the efficient progression of the wound healing process. Lipids play a significant role in balancing immune responses, underscoring their crucial contribution to optimal tissue repair and recovery.

### 8.4. Lipid Signaling in Angiogenesis

Angiogenesis, the formation of new blood vessels, is a highly complicated process that may be crucial for wound healing. One commonly held belief is that wound healing requires a robust and active angiogenic response [107,108]. However, several studies have demonstrated that skin wound closure can proceed normally even when angiogenesis is reduced [109,110]. This result suggests that while angiogenesis is typically associated with the wound healing process, an excessive or exaggerated response might not be beneficial and could even be unnecessary [111,112]. There are a number of significant lipid players and their signaling pathways that are essential for the complex choreography of angiogenesis. S1P, a bioactive sphingolipid generated through the sphingosine kinase-mediated phosphorylation of sphingosine, influences endothelial cell migration and proliferation, as well as vascular permeability. Using full-thickness splinted wounds on the back of sphingosine kinase-1 knockout mice, Aoki and coworkers demonstrated that S1P increases angiogenesis and the recruitment of T cells and macrophages, thus accelerating wound healing [113]. S1P modulates angiogenesis through its interaction with the S1P receptors, S1PR1 and S1PR3, found on vascular endothelial cells, prompting the formation of capillary-like networks [114]. Additionally, S1P enhances adherens junction assembly in endothelial cells, leading to a robust inhibition of VEGF-induced endothelial cell transmonolayer permeability in vitro as well as vascular permeability in vivo in mice [115]. Given the elevated levels of S1P in the bloodstream as compared to other tissues [116], this vascular permeability-regulating function of S1P aids in upholding the endothelial barrier integrity in specific vascular beds, a process mediated by endothelial cell S1PR1 [117]. S1P also exerts anti-angiogenic effects upon binding to S1PR2, expressed in bone-marrow-derived cells, reflecting the complex role of S1P in angiogenesis [118]. Also, the binding of S1P to S1PR2 disrupts endothelial barrier permeability [119].

PGE_2_ plays a multifaceted role in angiogenesis by promoting endothelial cell proliferation, migration, and vascular tube formation [120]. PGE_2_ achieves these effects through its interaction with specific G-protein-coupled receptors, influencing downstream signaling cascades [121].

### 8.5. Lipids in Keratinocyte and Fibroblast Migration and Proliferation

The coordinated actions of migrating and proliferating keratinocytes and fibroblasts are essential for tissue regeneration, wound closure, and the restoration of skin integrity, ensuring a successful and efficient healing process. The involvement of lipids in keratinocyte migration and proliferation is multifaceted, and various lipid mediators contribute to the course of wound healing.

Diacylglycerol, a well-known lipid signal, can be generated by the action of phospholipase C on cellular phospholipids. Diacylglycerol serves as an activator of several members of the protein kinase C (PKC) family of protein kinases. Many of these PKC isoenzymes are expressed in the skin and several are known to be involved in regulating keratinocyte proliferation and/or differentiation [122,123]. Diacylglycerol also activates protein kinase D (PKD), for which the evidence indicates a pro-proliferative effect [124,125,126]. Diacylglycerol can also be produced by the lipin-mediated dephosphorylation of the phosphatidic acid produced by phospholipase D activity [127,128]. On the other hand, phosphatidic acid can itself also serve as a lipid signal, with its own set of effector enzymes [127].

S1P and lysophosphatidic acid (LPA), another bioactive lipid that can be generated by the diacylation of phosphatidic acid, play a role in both keratinocyte and fibroblast migration and proliferation, contributing to wound healing processes [129,130,131]. Ceramides, a class of sphingolipids, are also implicated in regulating keratinocyte migration and proliferation during wound healing [132,133]. In a recent investigation, a ceramide kinase (CERK) inhibitor, SYR382141, was used to demonstrate that a reduction in CERK-generated C1P levels contributes to an expedited healing process in skin wounds [134]. The acceleration of wound healing through CERK inhibition with SYR382141 appeared to be linked, at least partially, to observed increases in fibroblast activation protein expression, the influx of infiltrating cells, and the deposition of type 1 collagen [134]. These findings suggest that inhibiting the formation of CERK-derived C1P may prompt a shift from the inflammatory stage to the proliferation phase, a crucial transition for effective wound healing [135].

Our work with phosphatidylglycerol suggests that different species of PG have different effects on keratinocyte proliferation, with PG species containing saturated and monosaturated fatty acids stimulating the proliferation of slowly growing keratinocytes and PG types possessing polyunsaturated fatty acids inhibiting the growth of rapidly proliferating cells [136]. Understanding how different PGs, as well as other lipids, affect keratinocyte proliferation is relevant to comprehending their potential roles in the overall process of wound healing and skin regeneration.

Prostaglandins are key regulators of the early stages of fibroblast and keratinocyte proliferation. PGE_2_, primarily produced by immigrating macrophages and stromal cells, serves as a stimulator for both angiogenesis and fibroblast proliferation [137,138]. Its influence extends beyond the immediate wound environment, highlighting its importance in coordinating cellular responses during the wound healing process. Importantly, prostaglandin D_2_ is involved in limiting fibroblast migration, preventing the undesirable outcome of excessive fibrosis or keloid formation [139]. This dual role of prostaglandins highlights the nuanced regulatory functions of lipids in modulating fibroblast behavior.

In summary, lipids serve as integral signaling molecules in the intricate dance of keratinocyte and fibroblast proliferation and migration during the wound healing proliferation phase, ensuring a finely tuned choreography of cellular events that contribute to effective tissue repair while minimizing fibrotic outcomes. A detailed examination of lipid functions is provided in Table 3, which offers a comprehensive overview of how different lipids contribute to the wound healing process.

### 8.6. Lipid Signals in the Remodeling Phase of Wound Healing

The remodeling phase of skin wound healing is a critical stage characterized by the maturation and restructuring of the ECM to restore tissue integrity. After fibroblast proliferation, the synthesis and deposition of collagen plays a crucial role in replacing damaged connective tissue. This process is initiated by the activation of fibroblasts, which later differentiate into alpha-smooth muscle actin-positive myofibroblasts induced by TGFβ signaling. One class of lipid signaling molecules that play a crucial role in the remodeling phase is the eicosanoids. Prostaglandins, such as PGE_2_, act as an antagonist of TGFβ signaling to limit fibrosis [148], playing a crucial role in the remodeling phase of wound healing. PGE_2_ regulates MMPs and TIMPs, balancing ECM turnover and inhibiting TGFβ1-induced collagen synthesis in dermal fibroblasts, ultimately reducing the risk of hypertrophic scar formation and promoting the formation of functional scar tissue [147]. Conversely, leukotrienes LTB_4_ and LTC_4_ have been associated with the aberrant activation of fibroblasts and fibrosis [144,145]. The controlled balance between pro-fibrotic and anti-fibrotic lipid signals is crucial for optimal tissue remodeling.

SPMs, including resolvins and lipoxins, also contribute to the regulation of the remodeling phase. Resolvin D2 (RvD2) has been demonstrated to inhibit TGFβ-induced fibroblast proliferation and migration, suggesting its role in modulating fibrosis during tissue remodeling [143]. Lipoxin A_4_, derived from arachidonic acid, has been associated with enhanced fibroblast proliferation and migration in vitro, suggesting its potential anti-fibrotic properties during tissue remodeling [143]. Additionally, lipid signals are involved in the regulation of collagen synthesis and ECM deposition, crucial aspects of the remodeling phase [149,150]. The orchestrated actions of eicosanoids, SPMs, and phospholipids contribute to the balance between fibrotic and anti-fibrotic responses, ensuring proper ECM remodeling and tissue maturation. To gain a comprehensive understanding of the intricate role of lipid mediators in regulating wound healing signaling during aging and senescence, exploring additional reviews, including the comprehensive analysis by Pils et al. [151], is recommended.

In summary, lipid signals play a multifaceted role in the remodeling phase of skin wound healing. From influencing fibroblast behavior to modulating inflammation and collagen deposition, these lipid signals contribute to the finely tuned organization of events that culminate in the restoration of tissue integrity. The delicate balance maintained by various lipid mediators ensures effective tissue remodeling while minimizing fibrotic outcomes, highlighting the intricate regulatory roles of lipids in the wound healing process.

## 9. Conclusions

In conclusion, the investigation of skin wound healing models allows an understanding of the role of lipids in the skin, emphasizing their crucial involvement in cellular signaling and tissue repair processes. Through meticulous investigation and experimentation, researchers have unveiled the multifaceted roles of lipids, from serving as structural components of cell membranes to orchestrating complex signaling cascades that regulate inflammation, proliferation, and tissue remodeling. The findings discussed here highlight the potential therapeutic implications of modulating lipid pathways to enhance wound healing outcomes. Further research is needed to elucidate the specific functions and interactions of different lipid species within the wound microenvironment. Targeting lipid-mediated processes offers a promising avenue for innovative wound care strategies, necessitating interdisciplinary collaboration and advanced technologies for comprehensive understanding and clinical translation. These insights may lead to personalized interventions and improved outcomes in wound healing and tissue regeneration.

## Figures and Tables

**Table 1:** Comparison of in vitro models for cutaneous wound healing studies.

In Vitro Model Type	Description	Advantages	Disadvantages
Single-cell-type 2D Models	Single-cell types cultured to investigate basic cell signaling responses to injury and stress, typically created by “scratch wounding” techniques.	Simple and cost-effective. Easy to manipulate and control experimental conditions. Provide valuable insights into basic cellular responses to injury.	Lack complexity of tissue microenvironment. Limited representation of cellular interactions and signaling pathways. May not fully replicate in vivo wound healing processes.
Co-culture Systems	Different cell types cultured together to investigate interactions and responses to injury; may be facilitated by Transwell systems for analyzing paracrine factors and/or chemo-tactic responses.	Allow for studying cell–cell interactions. Mimic paracrine signaling between different cell types. Relatively simple to set up and conduct experiments.	May not fully replicate the complex environment of tissue. Limited representation of in vivo wound healing dynamics. Require careful optimization of culture conditions.
3D In Vitro Models	Tissue architecture designed to replicate the physiological complexity of skin tissue, allowing assessment of wound contraction, migration, and matrix compaction in a 3D environment.	Improved simulation of tissue architecture and cellular interactions. Provide a more physiologically relevant environment. Allow for studying cell behavior in a 3D context.	More complex to establish and maintain. Require specialized equipment and expertise. Limited scalability for high-throughput experiments.
3D Skin Equivalents	Advanced models incorporat- ing multiple cell types and lay-ers to mimic native skin architecture, providing insights into tissue regeneration and re-epithelialization.	Closest representation of native skin architecture and function. Allow for studying multiple cell types and their interactions. Can incorporate ECM components for better simulation of tissue microenvironment.	More expensive and time-consuming to develop. Require advanced tissue engineering techniques. May lack full representation of immune response and vasculature.
3D Bioprinting	Constructed patient-specific skin grafts with biomimetic structures.	Enables precise control over tissue architecture and composition. Allows for the creation of patient-specific constructs. Offers potential for person-alized medicine and tissue engineering applications.	Limited by current technology in terms of complexity and scale. Challenges in achieving full functional integration of printed tissues. Costly and requires specialized equipment and materials.
Microfluidic Plat-forms	Microchannel designs to create cell-free wound areas for studying molecular processes in wound healing, including cell migration and interactions.	Provide precise control over microenvironment and cell–cell interactions. Enable real-time imaging and analysis of cellular processes. Offer potential for high-throughput screening and personalized medicine.	Require expertise in microfabrication and microfluidics. Limited representation of tissue architecture and complexity. Challenges in integrating with conventional cell culture techniques.
Ex Vivo Models	Living tissue samples harvested from organisms and cultured to study wound repair mech-anisms.	Close representation of native skin architecture and function maintaining cell–cell interactions. Allow for studying tissue responses in a more physiologically relevant context. Provide valuable insights into wound healing mechanisms.	Limited by tissue availability and viability. Require careful handling and maintenance of tissue samples. Lack dynamic aspects of in vivo wound healing environment.

**Table 2:** Comparison of in vivo models for cutaneous wound healing studies.

Model Type	Description	Advantages	Disadvantages
Rodent Models			
a. Full-thickness wound model	Wounds created that pene-trate through the entire thickness of the skin in rodents.	Allows for the study of wound healing processes in a controlled manner. Mimics human wound healing process, allowing for comprehensive assessment of healing dynamics.	Healing mechanisms may differ from human wounds due to anatomical and physiological differences.
b. Splinted full-thickness wound model	Similar to the full-thickness wound model, but involves the use of a splint to prevent wound contraction, allowing for granulation tissue formation and healing by re-epithelialization.	Provides controlled wound environment, minimizing wound contraction. Facilitates accurate assessment of wound closure. More accurately mimics human skin wound healing.	Requires additional equipment and technical expertise for splint application.
c. Tail excisional wound model	Similar to the full-thickness wound model, but wounds are created on the tail of rodents.	Convenient and easily accessible wound site for observation. Healing primarily through re-epithelialization like human skin wounds. Good for studying delayed wound healing.	Limited wound size and healing characteristics. Differences in anatomical sites compared to larger wound models.
d. Chronic wound models	Models created produce chronic wounds, such as diabetic ulcers or pressure ulcers.	Facilitates the study of long-term wound healing. Particularly relevant for chronic wound conditions.	May require specialized induction methods. Longer observation periods, increasing study complexity and duration.
e. Genetically modified (transgenic) models	Models created in rodents with specific genetic alterations to study the role of particular genes in wound healing.	Permits investigation of specific molecular pathways.	Genetic modifications may not fully represent human genetic conditions. Wound healing responses may vary.
f. Immuno-compromised model	Models created in rodents with compromised immune systems to study wound healing in the absence of immune responses.	Facilitates the study of the immune system’s role in wound healing. Minimizes interference from the host’s immune response.	Increased susceptibility to infections. Altered wound healing responses compared to immunocompetent models.
Other Models			
Rabbit Ear Model	Wounds created in the thin skin of rabbit ears.	Mimics certain aspects of human skin anatomy, such as thickness and vascularity, more accurately than rodent models. Provides a larger wound site for observation and standardized wound creation compared to rodent models.	Skin thickness, hair patterns, and immune responses differ from humans. Direct translation of findings may be limited.
Pig Skin Model	Wounds created in pig skin for wound healing studies.	Closest to human skin in terms of anatomy, thick- ness, and healing mechanisms among animal models. Suitable for studying wound healing in larger, deeper wounds that better simulate human injuries.	Higher cost and maintenance compared to rodent and rabbit models. Limited availability of specific strains or genetically modified pigs for research purposes.
Superficial or tape stripping model	Superficial layers of the skin removed using tape strip-ping.	Allows for investigation of skin barrier function and permeability repair in a controlled manner. Mimics certain aspects of wound healing without the need for invasive procedures.	Limited to superficial wounds. May not replicate complexities of deep wounds.

**Table 3:** Role and mechanisms of action of lipids in the skin wound healing process.

Lipid	Role and/or Skin Wound Healing Phase	Effect/Mechanism of Action of Wound Healing
Ceramide-1-phosphate (C1P)	Skin barrier maintenance	Maintains acidic pH to inhibit pathogenic microorganism growth [79]. Promotes β-defensin 2 expression, a vital antimicrobial peptide in human skin [80].
	Promotion of inflammation	Mediates inflammation through stimulation of cytosolic phospholipase A_2_ and the subsequent release of arachidonic acid and prostaglandin formation [96]. Serves as a checkpoint for phase transition from the inflammation to the proliferation phase [134,135].
	Proliferation	Inhibits keratinocyte proliferation [134]. Plays a role in orderly migration of fibroblasts in the wound site [140].
Cholesterol	Skin barrier maintenance	Maintains skin barrier and integrity [141].
	Promotion of inflammation	Contributes to lipid raft synthesis, facilitating assembly of inflammation pathway signaling molecules [77,78].
	Resolution of inflammation	Modulates inflammation by synthesizing steroid hormones like glucocorticoids, which regulate immune responses and control the intensity and duration of inflammation at the wound site [77].
Diacylglycerol	Proliferation phase	Activates protein kinases C and D, regulating keratinocyte proliferation and differentiation [122,123,124].
Omega-3 polyunsaturated fatty acids (PUFAs)	Promotion of inflammation	Increase proinflammatory cytokine IL-1β levels and prolong wound closure time in healthy human skin (marine-derived omega-3 fatty acids) [97].
	Resolution of inflammation	Topical DHA treatment accelerates rat skin wound healing by activating GPR120, which reduces IL-1β expression and increases IL-6 levels [101]. Intraperitoneal administration of a fish-oil emulsion rich in EPA and DHA improved excisional skin wound healing in diabetic male rats [102].
	Remodeling	Reduced wound strength due to changes in the fibroblastic or remodeling phases of wound healing [98].
Sphingosine-1-phosphate (S1P)	Promotion of inflammation	Promotes inflammation and regulates immune cell trafficking, vascular permeability, and pro-inflammatory cytokine production [95,129]. Increases the recruitment of T cells and macrophages [113].
	Resolution of inflammation	Modulates immune cell trafficking in inflamed tissues, promoting T cell retention (through S1PR1 and S1PR5) [142]. Promotes angiogenesis by enhancing the migration and sprouting of endothelial cells [113].
	Proliferation	Promotes keratinocyte and fibroblast proliferation and migration [131].
Specialized Pro-resolving Mediators (SPMs) such as Resolvin D2 (RvD2) and Lipoxin A4	Resolution of inflammation	Dampen inflammation and promote wound healing by reducing the production of pro-inflammatory cytokines [87,99]. Inhibit neutrophil infiltration, enhancing macrophage phagocytosis of apoptotic cells [99].
	Proliferation	Inhibit fibroblast proliferation and migration [143].
	Remodeling	Modulate fibrosis during tissue remodeling [143].
Leukotrienes	Promotion of inflammation	Serve as chemoattractants, aiding in wound healing by recruiting white blood cells [90,91].
	Proliferation	LTB_4_ and LTC_4_ cause aberrant activation of fibroblasts, collagen synthesis, and fibrosis [144,145].
Phosphatidylglycerol (PG or DOPG)	Inflammation	Facilitates skin and corneal wound healing [104,105]. Dampens inflammation by inhibiting TLR2/4 pathway activation [146].
	Proliferation	Enhances keratinocyte proliferation [136].
Platelet-Activating Factor (PAF)	Promotion of inflammation	Promotes immune cell recruitment, enhancing leukocyte adhesion and extravasation, release of proinflammatory mediators, and vascular permeability [106].
	Resolution of inflammation	Promotes apoptosis of neutrophils and clearance of inflammatory cells and debris and regulates pro-inflammatory mediators [106].
Prostaglandin E_2_ (PGE_2_)	Promotion of inflammation	Contributes to vasodilation and increased vascular permeability and facilitates immune cell recruitment [87,138].
	Resolution of inflammation	Stimulates angiogenesis and inhibits production of pro-inflammatory cytokines and leukotrienes [138].
	Proliferation	Promotes fibroblast and keratinocyte proliferation and migration [137].
	Remodeling	Inhibits TGFβ1-induced collagen synthesis and reduces hypertrophic scar formation [147].
Prostaglandin D_2_ (PGD_2_)	Proliferation	Regulates/inhibits fibroblast migration [139].
	Remodeling	Regulates tissue regeneration preventing fibrosis or keloids [139].
Thromboxane A2 (TXA2)	Promotion of hemostasis	Amplifies platelet activation and irreversible aggregation for hemostasis [82].
	Promotion of inflammation	Induces the synthesis of pro-inflammatory cytokine intereukin-6 and PGE_2_ [85]. Inhibits the expression of the anti-inflammatory macrophage marker CD206 [85].
Lysophosphatidic Acid (LPA)	Proliferation	Promotes fibroblast migration and proliferation [129,130,131].
